# Seasonal Pattern of Lesion Development in Diseased *Fraxinus excelsior* Infected by *Hymenoscyphus pseudoalbidus*

**DOI:** 10.1371/journal.pone.0076429

**Published:** 2014-04-23

**Authors:** Stina Barbro Katrin Bengtsson, Pia Barklund, Claudia von Brömssen, Jan Stenlid

**Affiliations:** 1 Uppsala BioCenter, Department of Forest Mycology and Plant Pathology, Swedish University of Agricultural Sciences, Uppsala, Sweden; 2 Department of Economics, Swedish University of Agricultural Sciences, Uppsala, Sweden; Virginia Tech, United States of America

## Abstract

Ash dieback is a recent widespread disease on ash (*Fraxinus* sp.) that is causing important economic and ecological losses throughout Europe. The disease is initiated by the ascomycetous fungus *Hymenoscyphus pseudoalbidus* (anamorph *Chalara fraxinea*). The main aim of this study was to investigate seasonal pattern of lesion development associated with ash dieback. We present data on the spread of 324 natural lesions in ash shoots, branches and stems surveyed over a 32 month period. Most lesions were active and showed the greatest rate of growth during the summer; however, lesions were active throughout the year. Tree mortality was high, with more than a third of the surveyed trees dying during the study. Although many lesions permanently ceased to develop, the rate at which new lesions emerged was greater than the rate at which lesions entered a resting phase. The most common cause for a lesion going into a permanent state of rest was that it had encountered a branch-base. Genotype analysis showed that multiple infections can occur in a single tree given that different genotypes were identified in different lesions as well as in single lesions. A weak positive correlation was noted between tree health and tree size and a weak negative correlation was noted between tree overall health and lesion activity. The lower limit for *H. pseudoalbidus* growth in culture was between 4.0°C and 0.5°C.

## Introduction

Ash trees (*Fraxinus* sp.) in Europe are threatened by the ash dieback epidemic. The fungus *Hymenoscyphus pseudoalbidus* (anamorph *Chalara fraxinea*) is the pathogenic agent of the disease [1]–[3]. In 2010, the Swedish Species Information Centre placed European ash (*Fraxinus excelsior*) on the list of threatened species [4], and *C. fraxinea* was placed on the European and Mediterranean Plant Protection Organization (EPPO) Alert List in September 2007 [5].

Based on the absence of a substantial part of the genetic variation shown by microsatellite markers, it has been hypothesised that the fungus is a recently introduced species [6], [7]. The subsequent discovery that the fungus existed in Japan before the onset of the European disease outbreak and that Japanese populations have a higher genetic variation further supports the idea that the fungus has been introduced [8].

Symptoms of the disease include wilting as a result of dying shoots and discoloration of different parts of the tree: brown spots on buds, leaves and leafstalks; dead shoots; and brown lesions on branches and stems. In severely diseased trees, *H. pseudoalbidus* has also been found in roots [9]. Tree crown shape changes as new shoots replace dead shoots, combined with disturbance of the apical dominance, resulting in a bushy appearance of the crown [10]. The first symptoms appear in the younger parts of the tree [10] and the fungus can be found in the leaves and petioles [11], [12]. The fungus forms appressoria on leaves and petioles and enters the tree directly through the epidermal cells [13]. Once inside, the fungus spreads effectively in all directions and in all tissue types of ash tree [9]. Schumacher et al. (2010) demonstrated that the bark lesions correlate well with the brownish discoloration of the wood [9], although the discoloration tends to be more extensive than the bark lesion. It has been suggested that the phytotoxin viridiol produced by *H. pseudoalbidus* could be involved in disease development because it can provoke brown necrotic spots on ash leaves [14], [15].

A few factors that influence the disease development of ash dieback have been identified. For example, the genetics of the ash tree are important given that studies have shown that some clones are more resistant than others. However, none of the clones that have been investigated were completely free from disease [16]–[19]. Ash clones that are susceptible to *H. pseudoalbidus* have a prolonged growing season in the autumn [18],[19], which hypothetically extends the period in which the pathogen could infect and establish in the tree. It has also been shown that small trees are more severely affected than bigger trees [10], [18].

Stand characteristics can have a significant effect on the disease severity in thinned stands and in suppressed trees [11]. Moreover, Kenigsvalde et al. (2010) showed that wet areas were associated with greater disease severity [20].

The fungus may be a source of the variation seen in disease symptoms: Bakys et al. (2009) observed a variation in the virulence of fungal genotypes; however, it was not possible to distinguish the influence of the strain from effect of the tree genotype [1]. Moreover, Kowalski and Bartnik (2010) reported a variation in growth rate between cultured fungal isolates from different regions [21].

The apothecia of *H. pseudoalbidus* develop on the previous year's petioles in the forest litter and ascospores are released from the end of June to the end of September in Norway [22], where ash leaves sprout in May and June and fall in September and October.

The goal of this study was to reveal the seasonal pattern of disease development for ash dieback by observing the activity of individual lesions. The observed pattern was analysed by comparing lesion development with different factors such as month, locality, year and position of the lesion. The results provide a contribution to the description of the disease-cycle of the ash dieback epidemic.

## Material & Methods

### Survey of naturally infected ash trees

Two-hundred and sixty-one naturally infected ash trees were surveyed from spring 2008 until autumn 2010. The trees were situated in middle-east Sweden at three sites (Gnesta, latitude 59.14332, longitude 17.21086; Österbybruk, latitude 60.28205, longitude 17.94514; Åkersberga, latitude 59.55764, longitude 18.57994) and at Ultuna (latitude 59.81488, longitude 17.65821). The temperature reference data from existing weather stations data did not differ significantly between the different weather stations in the surveyed area, only data from the Ultuna weather station was used in the final analysis. The trees at Gnesta and Österbybruk were planted in 1992 and 1994, respectively, but varied considerably in height (between one and ten metres). At Åkersberga, trees between one and ten metres in height were studied in a naturally regenerated stand dominated by ash. Hereafter, the Gnesta, Österbybruk and Åkersberga sites are collectively referred to as 'the three sites'. The 52 seedlings surveyed at Ultuna were planted in September 2007 and about 1.3 m tall.

The diseased trees were characterised by lesions on branches and stems. The lesion was the necrotic inner bark visible through the bark, which made it possible to record the development of the colonisation of the pathogen.

Lesion development was surveyed once a month. Besides noting if a lesion had expanded since the last measurement *i.e.* was “active” or not, the area of spread in cm^2^ (used to determine lesion growth rate) as well as the borders showing the least and most growth since the previous month were recorded. A lesion was considered to be permanently resting when at least 12 months had passed since the last recorded activity.

Trees at 'the three sites' were selected with the aim to catch the variety of the stand, for example regarding soil-properties and water-logging (no data). For this purpose, all trees within three blocks at the planted stands in Gnesta and Österbybruk, were searched for an accessible lesion. In the naturally regenerated stand at Åkersberga, all trees along parallel transects were searched for accessible lesions. In January 2008, initially, one lesion per tree was selected at 'the three sites'. To increase the sample size, more trees were included in the study during the first year, by extending the size of the blocks and adding more transects. A total of 86, 118 and 144 trees were surveyed at Gnesta, Österbybruk and Åkersberga, respectively. The lesions were chosen based on accessibility from ground-level (up to 1.8 m above the ground), and were located in different parts of the tree (stem, branches with secondary shoots and primary shoots). For the numbers of lesions surveyed each month, see Table 1.

**Table 1:** Number of lesions at the four localities each month.

	Gnesta	Österbybruk	Åkersberga	Ultuna
feb-08	**16**	**14**	**15**	**10**
mar-08	**15**	**12**	**16**	**14**
apr-08	**18**	**29**	**33**	**19**
maj-08	**53**	**79**	**59**	**19**
jun-08	**48**	**76**	**48**	**23**
jul-08	**54**	**71**	**55**	**29**
aug-08	**63**	**84**	**69**	**26**
sep-08	**63**	**82**	**69**	**28**
okt-08	**61**	**82**	**71**	**30**
nov-08	**65**	**85**	**71**	**31**
dec-08	**66**	**85**	**70**	**36**
jan-09	**66**	**86**	**70**	**40**
feb-09	**66**	**86**	**70**	**41**
mar-09	**65**	**86**	**70**	**42**
apr-09	**61**	**83**	**65**	**44**
maj-09	**61**	**82**	**63**	**50**
jun-09	**57**	**80**	**59**	**52**
jul-09	**51**	**77**	**56**	**55**
aug-09	**49**	**75**	**56**	**55**
sep-09	**49**	**75**	**57**	**55**
okt-09	**47**	**75**	**54**	**53**
nov-09	**47**	**74**	**53**	**57**
dec-09	**46**	**74**	**52**	**71**
jan-10	**46**	**74**	**51**	**74**
feb-10	**44**	**74**	**51**	**76**
mar-10	**41**	**74**	**49**	**77**
apr-10	**41**	**74**	**49**	**73**
maj-10	**37**	**72**	**49**	**70**
jun-10	**30**	**60**	**46**	**48**
jul-10	**27**	**57**	**38**	**44**
aug-10	**27**	**54**	**36**	**44**
sep-10	**26**	**54**	**34**	**-**

At Gnesta, Österbybruk and Åkersberga one lesion per tree was surveyed, and additional trees were included in the survey during 2008. At Ultuna, all lesions were included in the survey as they emerged on the tree. Lesions were lost during the survey due to the death of the tree or a part of the tree due to another lesion girdling the branch or shoot. At Ultuna, lesions were also lost due to fusion of lesions.

The health status of the trees was assessed in summer 2009 and summer 2010 by estimating the proportion of healthy leaf-mass: For example a tree completely without leaves on one fourth of the shoots was given a 75% health status. Tree diameter at a height of 1.3 m (or right under the apical shoots if tree height was less than 1.3 m) and tree height were recorded. All measurements were made by the same person, to exclude bias.

At Ultuna, the ash seedlings were carefully searched for new lesions every month. Although all emerging lesions were surveyed, the number of 'new lesions per month' only included those that were more than 5 cm from an existing lesion so as to reduce the risk of confusing the extension of existing lesions with the emergence of a new lesion

The decrease in lesions between May 2010 and June 2010 seen in Ultuna was mainly ascribed to the fusion of neighbouring lesions (Table 1).

At the end of the survey, l69 lesions (95 samples from 'the three sites' and 69 samples from Ultuna) were analysed with species-specific primers [23]: Wood and bark pieces (1 cm) were removed from the margin of the lesions and DNA was extracted. The PCR reaction contained 10× PCR buffer, 0.2 mM dNTPs, 0.75 mM MgCl2, 0.3 U ThermoRed Taq polymerase, 0.02 µM of each primer, and 0.25 ng / µl DNA. PCR reaction were performed in a PC-960G gradient thermal cycler, TECTHUM lab, and the reaction started with 5 min of 95°C followed by 35 cycles of denaturation at 95°C for 30 s, annealing at 62°C for 30 s and extension at 72°C for 1 min, finalized with an extension step at 72°C for 8 min.

In addition, fungal outgrowth was isolated from a few inner bark and wood samples collected from all sites during the survey and from 66 samples from Ultuna at the end of the survey: Pieces of symptomatic ash material were surface sterilized with 2 min in 70% ethanol, 2 min in 3% sodium hypochlorite and again, 2 min of 70% ethanol. After rinsed in sterile water, 5 mm pieces of wood or inner-bark from the lesion margin were placed on 1% malt-agar-plates. During incubation at 20°C in darkness, outgrowth resembling reference cultures of *H. pseudoalbidus* was sub-cultured to new agar-plates.

Further, 145 lesions (25 from Gnesta, 38 from Österbybruk, 25 from Åkersberga and 57 from Ultuna) were dissected in order to compare the bark lesion border with the discoloration of the wood underneath. The bark and wood were peeled off with a sharp knife and the furthest extension of the lesion was measured.

We also investigated whether different genotypes of the pathogen were present in a single tree at the Ultuna site by analysing microsatellite markers [6]: Nine seedlings with two to five lesions each were sampled, by taking a 1 cm wood and bark piece from the margin of each lesion. The PCR reaction contained 10× PCR buffer, 0.2 mM dNTPs, 0.75 mM MgCl2, 0.5 U Dream Taq polymerase, 0.2 µM of each primer, and 0.25 ng / µl DNA. PCR reaction was performed in a Veriti 96 well Thermal Cycler, Applied Biosystems, and the reaction started with 5 min at 95°C, 35 cycles starting with 30 s at 56°C, 30 s at 72°C and ending with 30 s at 95°C, 1 min of 56°C and finally 30 min at 72°C. Fragment analysis was performed by Uppsala Genome Centre (http://www.igp.uu.se/Serviceverksamhet/Genomcenter/).

In the statistical analysis we model the probability for lesions to be active or not depending on the factors month, year, locality and position on the tree. The response variable is in this case a 0/1 variable (not active/active), hence we use a logistic regression model. Since we make several observations on each of the lesions we need to account for correlations between these non-independent observations and include therefore also a autoregressive error term in the model. The resulting model used is a generalised linear mixed model and is fitted using PROC GLIMMIX in the SAS Statistical software (V9.3, SAS Institute, Cary, NC, USA). We restricted the analysis to series that were complete or almost complete between May 2008 and September 2010.

Odds ratios are a relative measurement, where the odds for one factor level (e.g. one locality) are computed in relation to the odds for another factor level (another locality). In this paper we used odds ratios to give a value of how many times more likely one case is compared to another. All confidence intervals are calculated with a 0.05 significance level.

All necessary permits were obtained for the described study, which complied with all relevant regulations. The surveyed localities were private owned by Lars Edling, Andersbo (Österbybruk), Lars von Engeström, Heby slott, (Gnesta) and Östanå Fideikomiss Incorporated Company, Östanå gård (Åkersberga).

### Temperature optimum

Seventeen isolates of *H. pseudoalbidus*, with three replicates of each, were grown on 1% malt extract agar at five different temperatures: 25.0°C, 20.0°C, 12.0°C, 4.0°C and 0.5°C Growth in mm was measured twice in the month following inoculation within a month of incubation.

## Results

### Presence of H. pseudoalbidus

The presence of *H. pseudoalbidus* in the surveyed ash stands was confirmed by isolating the fungus from samples collected at each of the four sites during the period of the survey (data not shown). At the end of the survey many of the remaining lesions were sampled and tested with species-specific primers (Johansson et al. 2010). At Ultuna, 50 of 78 lesions were confirmed to be infected by *H. pseudoalbidus* with the use of species-specific primers (44 of 69) and/or by isolations (33 of 66). At 'the three sites', 16 of 95 lesions were confirmed to be infected by *H. pseudoalbidus* with the use of species-specific primers.

Different genotypes were found in each of the sampled lesions of nine seedlings that were investigated. In five samples, two alleles per microsatellite were recorded, which were interpreted as mixed genotypes (Table 2).

**Table 2:** Number of genotypes in nine investigated seedlings at Ultuna, distinguished by microsatellite markers.

Seedling	Sampled lesions	Samples with one genotype	Samples with several genotypes
U11	**2**	**2**	
U21	**2**	**1**	**1**
U22	**5**	**3**	**2**
U24	**3**	**3**	
U27	**2**	**2**	
U33	**2**	**2**	
U36	**2**	**2**	
U46	**3**	**3**	
U52	**4**	**2**	**2**

One sample per lesion was examined.

### Lesion development

The number of lesions that were surveyed decreased over time. A substantial number of the trees, 74 out of 261 (17.0%±8.1% per year), died during the survey (19 at Gnesta, 13 at Österbybruk, 36 at Åkersberga and six at Ultuna). Fifty-five of the 324 lesions were girdled by another lesion and, therefore, excluded from the survey. A few samples were taken for fungal isolation and a couple of trees were mechanically damaged. Although all the lesions on the seedlings at Ultuna were surveyed, a substantial proportion (38.3%) of the surveyed lesions was lost because of neighbouring lesions merging together. Notably, eight of the 52 Ultuna trees remained seemingly healthy and no lesions were observed during the period of the study.

The proportion of lesions that were active at least one month per year decreased over time. A lesion was defined as permanently resting if it had not been active for at least 12 months. Out of 247 lesions that were surveyed for at least 13 months, 104 became resting (25.3%±9.6% per year). In many cases, there was no visible cause to explain why lesion growth had ceased (27.0%±15.1% of all resting lesions), but generally resting coincided with the lesion reaching a branch base (50.0%±17.0%), or the junction of three or more shoots of equal size (15.4%±12.3%). In total, 42.0%±18.3% of all lesions situated at a branch base became resting per year. Two out of 88 lesions that stopped developing had been overgrown by bark and thereby sealed by the tree. Twelve of the lesions that ceased to develop for unknown reason, and two lesions that stopped at a junction of three shoots, resumed activity between 12 and 27 months later.

**Figure 1:**
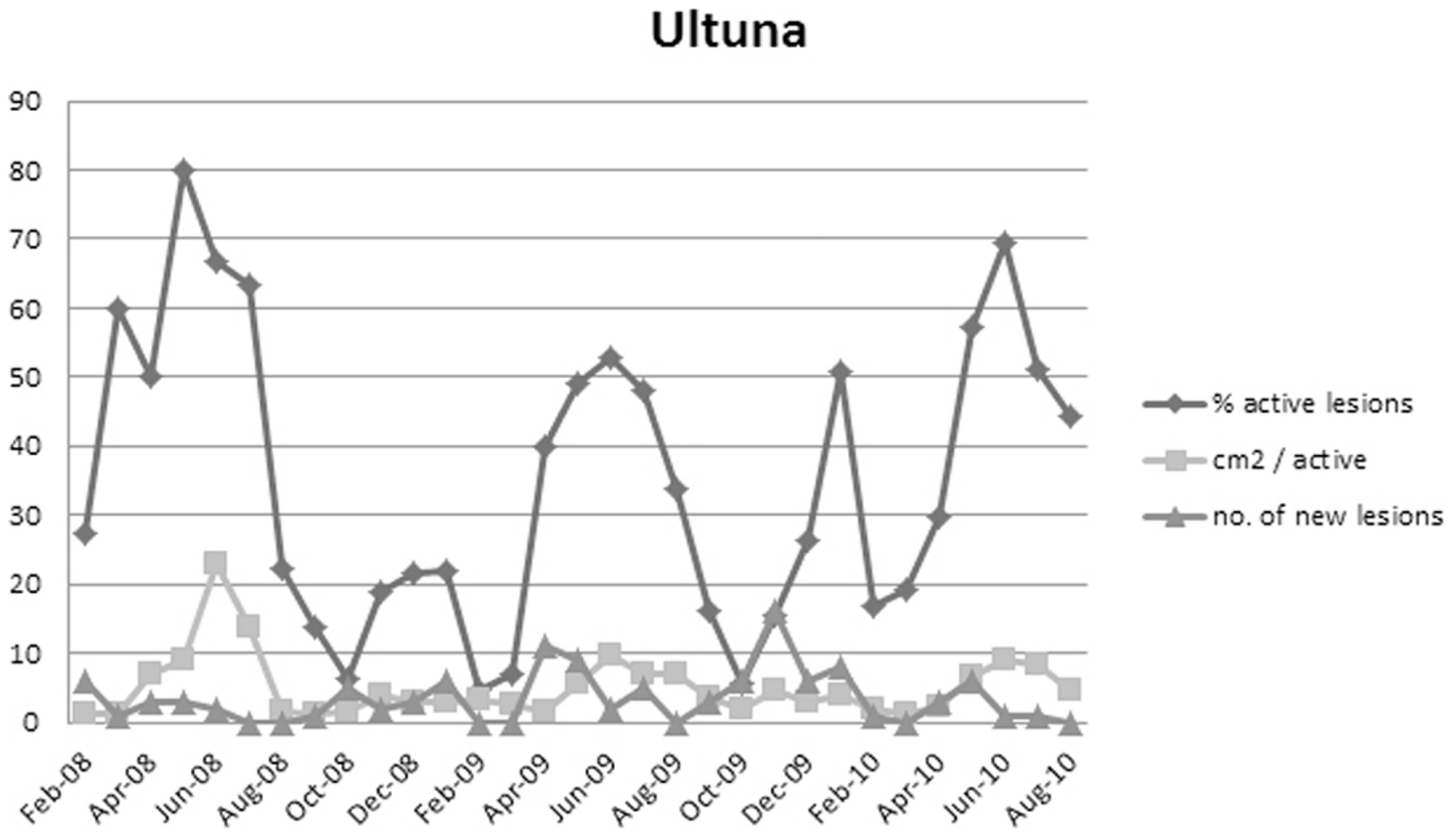
Lesion activity each month at Ultuna. Percentage of active lesions, growth rate measured as the sum of extension (cm^2^) of all active lesions each month divided by number of active lesions, and the number of new lesions emerging on the trees each month.

**Figure 2:**
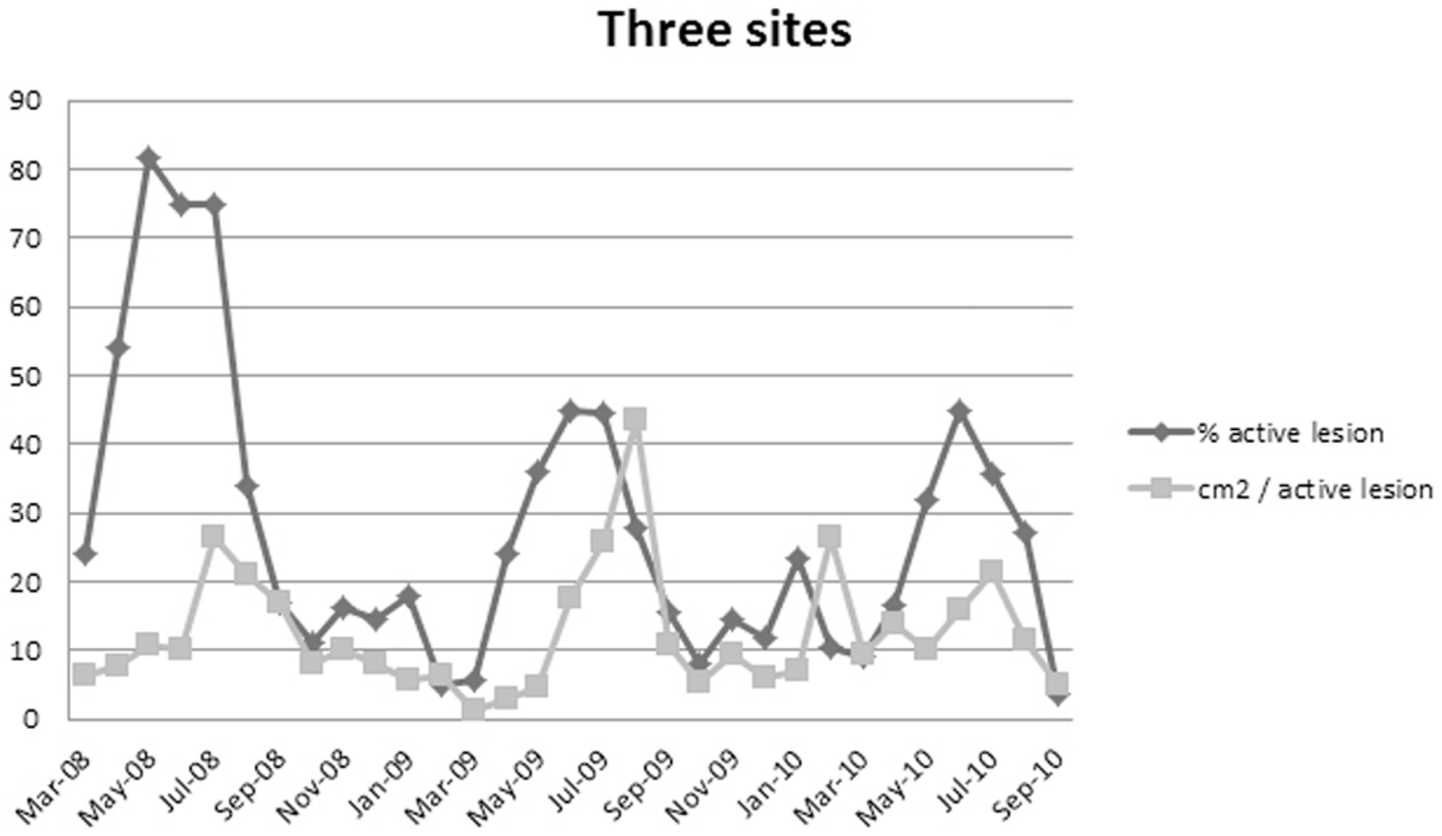
Lesion activity each month at 'the three sites'. Percentage of active lesions and the growth rate measured as the sum of extension (cm^2^) of all active lesions each month divided by number of active lesions.

**Figure 3:**
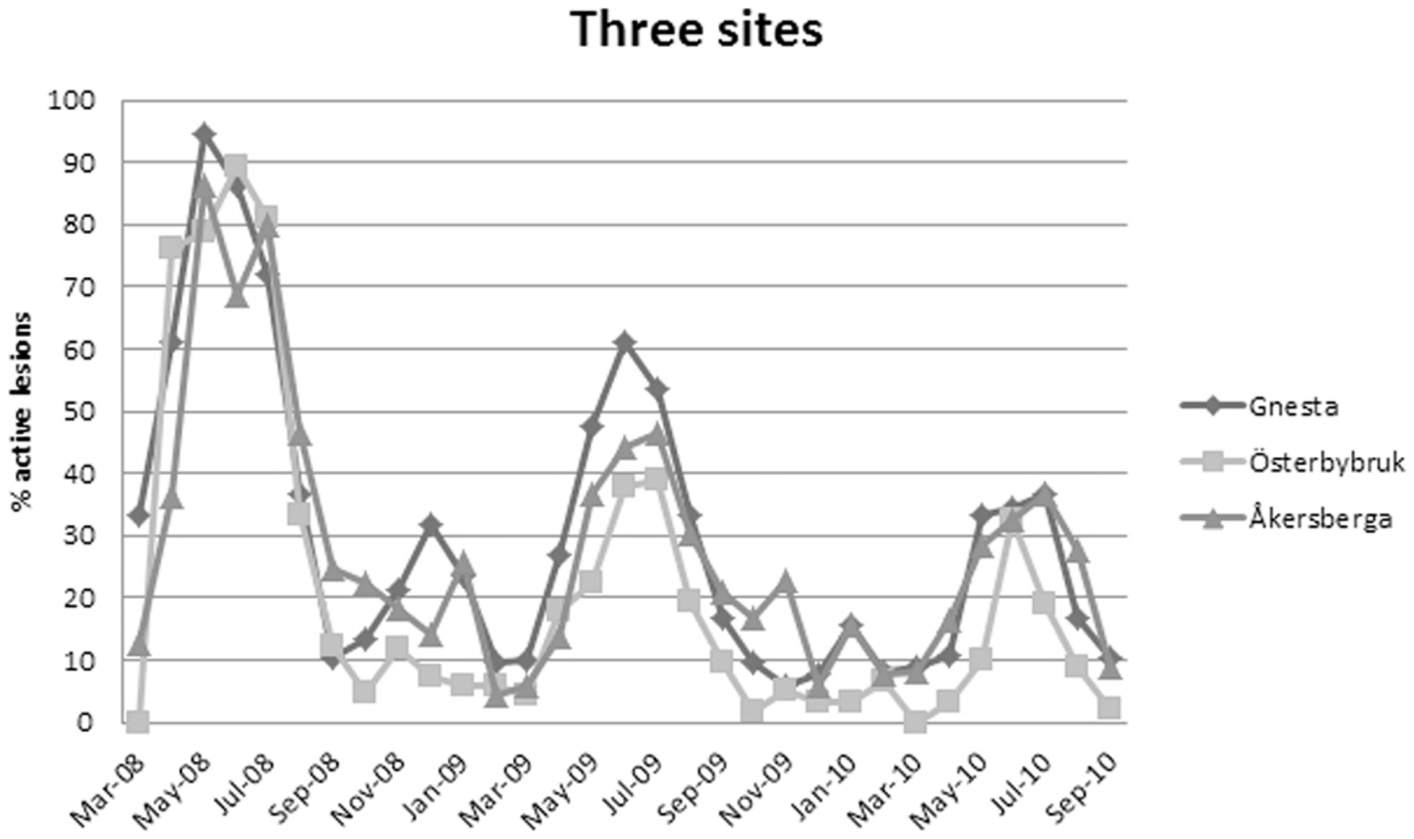
Percentage of active lesions each month at Gnesta, Österbybruk and Åkersberga.

The proportion of active lesions was highest in May (2008) and June (2009 and 2010), and a small peak was noted in January (Figures 1, 2 and 3). By contrast, the active lesions showed the greatest rate of growth in July (2008 and 2010) or August (2009) at 'the three sites', but in June at Ultuna. However, a small peak in the growth rate was observed at 'the three sites' in February 2010 (Figures 2 and 3). Using only the 86 lesions from 'the three sites' that were available between May 2008 and September 2010 in a logistic regression model, the number of active lesions was significantly greater between May and July compared with the rest of the year. Lesions in June were the most likely to be active (the odds were 19 times higher compared with those for December). Furthermore, the proportion of active lesions per month decreased with time for 'the three sites', with five times higher odds for lesion activity in 2008 compared with 2010, and two times higher odds in 2009 compared with 2010.

There was a locality-effect for 'the three sites', with the odds of a lesion actively developing at Gnesta 2.5 times higher than at Österbybruk. No significant difference was seen between Åkersberga and the two other sites (Figure 3).

The position of the lesion on the tree affected lesion development. Lesions situated on the stem had the highest odds of being active, almost five times higher than a lesion that had reached a branch base. There were also significantly higher odds for lesion activity on the stem compared with on old branches, or on young twigs or shoots compared with lesions on a branch base. As described above many lesions situated at a branch base became resting after a while, which will strongly influence this calculation.

At 'the three sites', a weak negative correlation was seen between tree health and the activity of the lesion (Pearson correlation −0.144, *p*<0.001). Furthermore, the size of the tree correlated positively with tree health (Pearson correlation 0.235, *p*<0.001). There was no visible correlation between tree size and lesion development. The health status of trees that died during the survey was significantly lower than of trees that did not die (T-test, *p*<0.05); however, seven trees that had been estimated to have at least 50% healthy leaf mass the previous year died the following year.

Temperature correlated positively with lesion activity (using cumulative temperature sum per month with a base level of 0°C, and percentage of active lesions). A linear regression analysis showed that temperature could explain 4.1% of the pattern for lesion activity at 'the three sites' (*p*<0.001). For Ultuna, 2.3% of lesion activity was explained by temperature (*p*<0.001). The correlation was a bit lower when comparing temperature with the growth rate of developing lesions: 2.6% for the three localities and 1.9% for Ultuna (*p*<0.001).

The temperature during January and February 2010 did not rise above 0°C. In spite of this, lesion activity occurred during this period.

**Figure 4:**
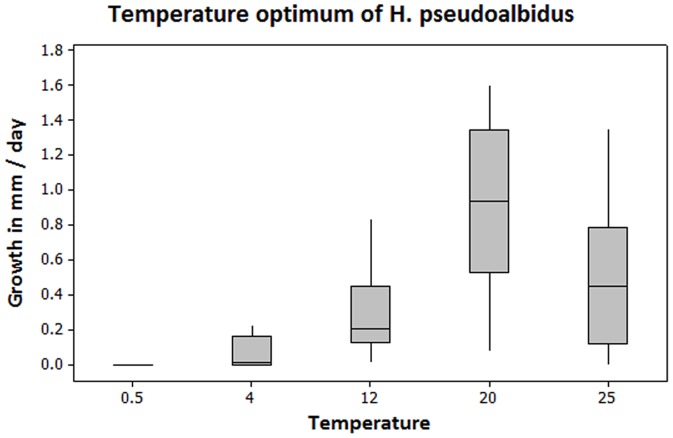
Growth rate (mm/day) at different temperatures for seventeen isolates cultured on malt-agar media.

The optimum temperature for mycelia growth in culture was 20.0°C. Furthermore, no mycelial growth occurred at 0.5°C, and six of seventeen isolates showed no growth at 4.0°C The variation in mycelia growth rate among isolates was large, but significantly different between all temperatures (*p*<0.01) (Figure 4).

### New lesions at Ultuna

New lesions emerged on the bark of seedlings growing at Ultuna throughout the year, (Figure 1). Each year, 42.6 new lesions emerged, or 0.82 per seedling a year. By contrast, only 7.4 lesions at Ultuna became permanently resting per year, or 0.14 per seedling a year.

Given that new lesions were only recorded once a month, the data are coarse. However, some of the originating positions could be documented. New lesions were detected on shoots, branches and stems with or without leaf-scars. In most cases (74.7%±17.4%) the lesion appeared in connection with a leaf-scar or bud. Ten of the 75 lesions appeared on the stem with no visible connection to a bud or a leaf-scar. In a few cases, the lesion emerged with no connection to a leaf-scar on a primary shoot, with a very low likelihood that it had originated from a previous lesion: for example, when the only existing lesion was situated on a distant shoot (four cases), or on a previously asymptomatic seedling (one case).

### Lesion vs. wood discoloration

The visible lesion was often connected with a more widely distributed discoloration in the wood: Out of 145 lesions, 67 lesions showed a greater area of discoloration in the wood (first quartile 2.0 cm; median 4.0 cm; third quartile 10.0 cm or at most 31 cm), and only one lesion extended further at the surface bark than in the wood. The area of wood discoloration also extended downward from the lesion border significantly more than upward (*p*<0.005).

Non-resting lesions differed significantly from resting lesions: 53.5%±14.4% lesions from the former group had more discoloration in the wood compared with the bark lesion (on average 3.9 cm more discoloration) whereas the latter group had only 29.5%±13.2% lesions with more discoloration in the wood compared with the bark lesion (*p*<0.05) (on average 1.5 cm more discoloration).

Resting lesions positioned at a branch-base showed significantly less wood discoloration than lesions that had stopped developing for unknown reasons: the former showed more wood discoloration in six out of 34 of the lesions (on average 0.9 cm), the latter in five out of seven lesions (on average 5.1 cm) (*p*>0.05, T-test). By contrast, wood discoloration did not differ between non-resting lesions and lesions that became resting for unknown reasons.

## Discussion

The data collected from all four localities showed a seasonal pattern for lesion activity, with lesion activity and growth rate peaking during the summer. Interestingly, the number of active lesions peaked a month before the lesion growth rate peaked for 'the three sites'. The delay might be an effect of differences in tree resource allocation due to month, as well as differences between trees regarding budburst and resistance [17]–[19]. The disparity might also be an effect of fungal developmental stage. More knowledge is needed about the defence mechanisms of the tree. Unlike at 'the three sites', at Ultuna the peak in lesion activity and growth rate appeared to coincide, possibly as a result of differences in the resource allocation and the resistance of young seedlings compared with the older trees at 'the three sites'.

Temperature could explain lesion activity to some degree. However, it was not possible to separate temperature from the mixed influence of the fungal and tree life-cycles, in this experimental set-up. Although more lesions per month were active at the most southerly site than at the most northerly site, no significant difference in mean temperature was observed between the sites (data not shown).

There was an annual decrease in lesion activity at 'the three sites', mainly explained by the increased proportion of permanently resting lesions. The fact that new lesions were continually being included at Ultuna partly explains the absence of such a pattern for this locality, sustaining a comparatively high proportion of non-resting lesions.

The presence of *H. pseudoalbidus* was confirmed in many of the lesions (64% at Ultuna but only 17% at 'the three sites'). The sampling was carried out at the end of the survey so as not to disturb lesion activity during the survey. Given that our results predict that about a fifth of the lesions will become permanently resting each year, more than half of all lesions would have been resting by the end of the survey. This might explain about three-quarters of the negative samples at 'the three sites' but leaves a quarter unexplained. We cannot completely reject the possibility that *H. pseudoalbidus* was not present in these lesions even though lesion activity had occurred during the same season. Inhibitory compounds in the DNA extract could also cause some of the negative results. As many as 56 fungal taxa have previously been recorded in ash shoots showing various disease conditions, and pathogenicity tests showed that three of these (*Alternaria alternata*, *Epicoccum nigrum* and Phomopsis sp.) cause necrosis on ash [1]. Moreover, Przybył (2002) showed that *Diplodia mutila* and *Fusarium solani* isolated from necrotic shoots of ash were pathogenic [24]. Regardless of whether *H. pseudoalbidus* was present in a specific lesion, several other fungi were present, and presumably more fungal species would be found in the older lesions. The role of *H. pseudoalbidus* as the main pathogenic agent of ash dieback disease has been confirmed by several studies [1]–[3]; however, the role of secondary fungi in combination with *H. pseudoalbidus* has not been thoroughly investigated, although Bakys et al. (2011) showed that the severity of ash dieback was increased in combination with root rot [25]. The interactions that occur in the fungal community within an ash lesion might result in various outcomes such as increased or decreased development of disease symptoms. Investigation of these interactions might lead to the detection of a candidate species for biological control of ash dieback.

It is intriguing that lesion development occurred during the coldest winter in the survey: the temperature did not rise above freezing point throughout January and February 2010. The optimal temperature for *H. pseudoalbidus* in culture is 20.0°C [21], and we showed that the fungus does not grow at 0.5°C in culture. However, the freezing point inside the tree tissue is reduced by, for example, sugar, which may allow fungal activity to still occur at lower ambient temperatures. Alternatively, and more likely, the January and February lesion activity peaks might reflect that the healthy tissue surrounding a lesion was more sensitive to frost damage during these sub-zero periods, possibly enhanced by frost bacteria [26]. Moreover, toxins or enzymes might spill over from the neighbouring dead cells, causing the development of a chemically induced lesion.

A relevant question is whether the new lesions represented new infections or if they were lesions that had spread under the bark. The position of the new lesion was often in association with a leaf-scar but not always. A considerable proportion of the lesions, where a starting position could be identified, appeared on the stem with no connection to a leaf-scar or damage. Lenticels are a possible entry point for many fungi and might be for *H. pseudoalbidus*, as suggested by Husson et al. (2012) [27]. Cleary et al. (2013) have shown that the fungus enters through the leaf cell wall, but they did not observe the fungus entering via the bark [13].

New lesions emerged throughout the year, seemingly more during spring and autumn. *H. pseudoalbidus* apothecia formation and ascospore release occurs during the summer [22]. Many new lesions emerged long after the period of ascospore release, which suggests that they were unlikely to be new infections. However, it is possible that the emergence of a lesion could still occur some time after spore infection: more knowledge about fungal latency periods is needed.

Schumacher et al. (2010) observed that the fungal hyphae spread well in the wood and reach out for the bark in a secondary manner [9]. In our survey, the wood discoloration could be as much as 31 cm from the visible lesion border. These observations further supports that new lesions did arrive from an old infection. However, in a few cases, new lesions emerged that had no connection to a leaf-scar and no likely source-lesion. In the few cases that this was observed, the lesion emerged on the bark of a primary shoot, which in some instances the fungus may be able to penetrate.

Even though we could not distinguish for certain between new infections and new lesions emerging from old lesions in this survey, we can predict what happens to an infected tree over time. We have shown the rate at which lesions became permanently resting; when combined with the rate of emergence of new lesions, these two factors indicate the likely outcome of the disease for the average tree. There is about an eight times greater chance that a seedling will gain a new lesion than lose the activity of an existing lesion. The variable susceptibility of individual trees, as well as site conditions will alter the outcome for individual trees. However, as a general measure it illustrates the high disease pressure in the investigated sites. Even though individual lesions become resting, new lesions will emerge, continually intensifying the effect of the disease.

We detected different genotypes of *H. pseudoalbidus* in the individual lesions of seedlings. This agrees with previous findings showing multiple infections in leaves and petioles [12], [13]. However, the finding of more than one allele per microsatellite marker in individual samples was more surprising. These samples may represent heterothallic strains of the fungus, although only homothallic isolates have been obtained from lesions in previous studies [6]. However, given that a high number of *H. pseudoalbidus* strains that have been shown to share the space of a single petiole or leaf [12], [13], it seems likely that several homothallic genotypes could exist in a 1 cm wood sample.

The position of a lesion seemed to strongly affect whether it would go into permanent rest or not given that almost half of the lesions positioned at a branch base became resting. The most common cause for a lesion to become resting was that it had reached a stem-base or a junction of three or more shoots of equal size. This strongly indicates an important effect of the tree's inactive physical defence.

The reason for a fifth of the lesions to become permanently resting was not obvious. However, based on dissection data, these lesions might have still been actively developing under the bark. A bigger sample size is needed to confirm this. Other factors such as competing secondary fungi or the tree's active defence mechanisms might also explain why some of the lesions ceased to develop.

More than a third of the trees died during the survey and only six out of 52 seedlings remained healthy. This level of disease resembles reports from European countries [20], [28]–[30] as well as disease severity in progeny trials [17]–[19], [31]. However, it is complicated to compare different stands of various tree ages and a changing proportion of susceptible versus less susceptible trees.

Previous studies have shown a positive effect of tree size on tree health. Even though the trees included in this study were all comparatively small, a weak effect of tree size on tree health was observed. Considering that trees from Gnesta and Österbybruk were all the same age, one might expect a stronger correlation at those sites due to variation in tree genetic susceptibility. However, it was observed that waterlogging and heavy clay often coincided with small trees, adding an important confounding environmental factor (data not shown), which is consistent with previous observations [20], complicating unequivocal interpretation.

The health status of the tree was hypothesized to correlate negatively with lesion activity, which was found to be the case, but to a very low extent. This effect might be due to genetic variation in tree susceptibility or be the result of reduced resources in a weakened tree. Some trees that looked reasonably healthy died a year later. There were many observations of stems being girdled by rapidly spreading lesions, killing the trees (data not shown). For trees of the sizes included in this survey, this could happen even though a substantial part of the leaf-mass remained. Additional fungal infection of single trees by, for example, *Armillaria* sp., may also increase tree mortality of diseased ash [32], [33].

This study was based on the measurement of visible lesion spread. However, by just observing the surface, the true level of fungal activity will always remain partly unknown. Bark lesions of ash dieback have been shown to correlate with wood discoloration [9]. However, the discoloration in the wood often exceeds the borders of the bark lesion [9]. In addition, Bakys et al. (2009) frequently isolated *H. pseudoalbidus* from asymptomatic petioles [1], and McKinney et al. (2012) detected *H. pseudoalbidus* DNA ten centimetres outside the lesion border [16]. Both surveys were performed in summer prior to leaf-fall, which is when most lesion activity occurs. These studies suggest that the observed lesion corresponds approximately, but not exactly, to the spread of *H. pseudoalbidus* in the tree. Therefore, lesion spread may have been somewhat underestimated in our survey. However, the essential pattern of lesion activity described here should not be affected.
